# The Value of the Examination of the Teeth in Anthropological Research

**Published:** 1890-12

**Authors:** H. Lloyd Williams


### SELECTIONS.

### The Value of the Examination of the Teeth in Anthropological Research.

BY H. LLOYD WILLIAMS, M.R.C.S., L.D.S.

The following article was almost entirely written as a critique upon Dr. Betty's paper* in the April number of the *Dental Review*. The subject is one which may prove of great importance to the scientific world generally, but it is certain that to the dental profession valuable facts can not fail to be discovered in such a research as Dr. Betty is pursuing in the Medical Museum at Washington.

In one part of my article a reference is made to Mexico as the country whence material, which may contribute largely to the solution of the question at issue—viz., the ancestry of the Indian tribes—may be found. Morton, when referring to the Toltecan family, which represented the civilization of Central America, refers to two or three conquests by nations less civilized, but who adopted the habits and customs of the vanquished, with possibly some modifications. The question arises—from whence did these nations migrate? Evidence already exists which points to the north or northwest as the direction from which at least one invasion came.

Further, the Anthropological Society of New York has published an account of explorations which were carried on in one of the southern states, where, after meeting with the skeletons and materials usually found in such excavations of Indian mounds, indications of another and deeper burial place were found; the discoveries here are not yet fully known, but the little that has been described concerning the place seems to point to a period anterior to the Indian mounds referred to in this article.

*"A Critical Examination of the Teeth of Several Races, Including 150 Mound-Builders, Selected from the Collection of the Army Medical Museum at Washington."—Dr. E. G. Betty, D.D.S., *Dental Review*.

It is a long time since the dental profession has had the pleasure of recording a continuous and vast research undertaken or completed by one of its busy members; the paper under notice betokens such a concentration of energy for a definite end as we have been strangers to for a long period.

The line of research followed lies in the direction taken by the late Mr. Mummery in his classical paper read many years ago before the Odontological Society of Great Britain on "The Relation of Dental Caries to Food and Social Condition."

In the latter paper it is sought, by direct and extensive examination of skulls to determine the effects of civilization, diet, and climatic conditions upon the teeth.

Following out these indications, the skulls are as clearly classified as the circumstances permitted, the antiquity of the skulls is discussed and proximately determined; the shape is also stated, and the habits of the people deduced from the nature of their implements, etc., where they are pre-historic, and from history or observation in the case of the more recent specimens. The tabulation has not the merit of detailed observation of each individual specimen, but it possesses another advantage which greatly enhances its value, viz., classification into distinct recognized races; the number of skulls belonging to each race examined is given, and the average for that number of cases of caries, irregularity, etc., recorded.

The paper written by Dr. Betty compares favorably with Mr. Mummery's paper in some important matters. Each individual specimen is precisely described in great detail, and it is here that the highest commendation is due to the writer. The information is given under the following headings: Race, Age, Sex, Lat. and Ant. Post. Diameters of Upper and Lower Jaws, Irregularity, Abscess, Caries, Calculus, Facial Angle, Remarks.

The selection of specimens for examination, so far as the published account has developed it, is open to objections. A series of 100 typical *skulls* without any regard to family or race was first taken, and secondly a series of the best *dentures* the museum afforded, again without regard to race. This will appear to every reader as a preparation for the task which Dr. Betty has undertaken, but it is a preparation which careful readers will not be disposed to neglect.

To derive any benefit from the published tables the reader must be a most accomplished ethnologist, for to find out the race or family a specimen belongs to will prove to be most perplexing. The reader is told that these matters have been disregarded because the books for their elucidation are at hand. That the books exist it is not disputed, but that one reader in fifty knows very much of them or possesses them for reference, or even dwells within reach of a library where such books can be obtained, we must be permitted to doubt. It will not take the writer a much longer time to consult authorities and classify his specimens intelligibly, while the gain to his readers will be almost incalculable. The term Indian is acknowledged to be a much abused one, and to merely state that the skull is that of a Hare, Clallum, Tesan, or ancient Pueblo Indian, does not really give us any reliable information regarding the original possessors. It is extremely improbable that even Americans would know to what great divison or stock the above specimens, with the exception of the last belonged, and the rest of the world probably lies in still greater darkness regarding them.

The necessity for such a detailed examination and classification as has been indicated is perceived when it is remembered that perhaps the greatest object to be attained is to observe the effects of the different phases of man's environments in his progress from barbarism to civilization upon his teeth.

In order to elucidate this department of human history it will be necessary to afford data of known value in order that new facts may receive due appreciation. The round headed (brachycephalic) skulls are recognized as denoting their possessors as a race intellectually more advanced than the long-headed dolichocephalic, and with a division of a number of skulls into these two classes a certain amount of intelligibility would be gained.

The third series of tables gives the result of the examination of 150 Mound-builders.

With regard to these Dr. Betty admits that their antiquity is a mooted point. A large number of the mounds which have been properly examined do not antedate the European invasion; a certain other number do not differ sufficiently from the former to warrant the conclusion that they are of much earlier origin.

The mounds of Tennessee and Kentucky may be cited as instances to show the difficulty of determining the value to be attached to the skulls that have been discovered in them. The Cherokee nation of Indians once occupied Carolina, Virginia, Tennessee, Kentucky, and Alabama; these territories were ceded to Europeans and secured by treaties from time to time. Many mounds have been opened in these parts, and a number of skulls from the Kentucky and Tennessee mounds are included in Dr. Betty's tables. The opinion expressed by those best qualified to judge concerning these mounds is that they are not ancient; in several, implements of distinctly European origin, such as sheet copper of different shapes, a sword, etc., have been discovered; others, although nothing could be found in them of European manufacture, depart so little in other characteristics from the former, that capable observers decline to give them a date much, if any, anterior to the time of the discovery of America.

Dr. Betty's further contributions will be looked forward to with eagerness, and we venture to hope that the next tables will contain the results of the examination of Mexican skulls. It is here possibly that the solution of the complicated question lies. Some light may be thrown upon the relationship of the Toltecan family to the American family of Morton, and thus establish an important link which will serve to elucidate much that is now concealed regarding the latter family. If a sufficient number of skulls of the Toltecan family can be obtained and classified as nearly as possible according to their relative positions in the scale of civilization, and a record of any thing in their history or in their country likely to bear upon the matter be added, a very important step will be have been taken in the right direction. Even if the determination of the relative positions of the nations comprised in the Toltecan family be the only result, that will be a great work. The determination of the ancestry of the Indians from the contents of the mounds already examined seems to be rather hopeless, still older burial places must exist somewhere, and there are already some indications of our being within reach of them.

Whether measurements of the lateral and antero-posterior diameters of the jaws, and observations on the teeth will ever form any but accessory aids to the anthropologist is as yet doubtful. Anthropologists do not give these measurements, and take comparatively little notice of the teeth. Dr. Betty seems to entertain the opinion that he may be able to solve, or at least contribute largely toward the solution of the question of the ancestry of the American Indian. The possibilities in this direction are entirely in his hands.

If, again, we view the results of the examinations of Dr. Betty as indications from which to deduce conclusions as to the diet, climatic and social conditions of the different tribes examined, and utilize the knowledge which Mr. Mummery's researches have placed at our disposal, we shall not find that much can be stated positively of the environment of a people from observations made upon their teeth.

A very striking fact which Mr. Mummery notices in his examination of British skulls illustrates the above contention. He found two cases of caries among sixty-eight Wiltshire skulls, whereas in about sixty-eight Yorkshire skulls he found twenty-six exhibiting disease. It is further of great interest to find that the Roman skulls in Yorkshire exhibited a much higher percentage of decay than in other parts of England. The ratio in other parts of England was thirty-two per cent.; those found in Yorkshire showed decay in eighteen out of twenty-three skulls.

The nature of the diet, whether animal, fish, or vegetable, or a combination of two or more of these, can be more readily determined. But in all cases where a statement as to the nature of the diet has been ventured upon, other circumstances, particularly the weapons found with or near the skulls, have been taken into consideration, and in this manner a sufficiently accurate knowledge can be obtained.

Dr. Betty is to be congratulated on the work which he has already executed, and we hope that as his work extends and his experience ripens, we shall obtain from him results of great importance.

THE FOLLOWING CAUTION from an Exchange should receive the thoughtful consideration of every one who uses chloroform.—*Ed.*

---

It seems to me that certain general facts or principles in regard to anæsthesia must be considered as established :

1. That the use of any anæsthetic is attended with an appreciable risk, and that no care will prevent an occasional loss of life.

2. That chloroform acts much more promptly and much more powerfully than ether, both upon the respiratory centers and the heart.

3. That the action of chloroform is much more persistent and permanent than that of ether.

4. That chloroform is capable of causing death either by primarily arresting the respiration, or by primarily stopping the heart, but that commonly both respiration and cardiac functions are abolished at about the same time.

5. That ether usually acts very much more powerfully upon the respiration than upon the circulation, but that occasionally, and especially when the heart is feeble, ether is capable of acting as a cardiac paralysant, and may produce death by cardiac arrest at a time when the respirations are fully maintained.

6. Chloroform kills as near as can be made out, proportionately three to five times as frequently as does ether, partly, no doubt, becase it is more powerful in depressing the heart, but largely because it lets go its hold much less rapidly than does ether when inhalation ceases. Is it not possible that this "holding on" is because it is less volatile than ether, and can we not here get a hint why chloroform is less deadly in the South than in the North? The diffusibility of vapors or gases is in inverse proportion to the square of their densities, and the vapor of chloroform would certainly diffuse itself with far greater rapidity at 90° F. than at 70° F.

---

DEATH FROM NITROUS OXIDE.—A death from nitrous oxide is reported from Montreal. A man, aged twenty-four, went to the office of a dentist to have a tooth extracted, and requested to have the gas administered. After assuring himself that the patient was not suffering from heart or lung disease, the dentist administered the gas. No sooner had the tooth been extracted than the patient gave a gasp and fell over in the chair. He was placed upon the floor and artificial respiration performed, but without restoring animation. The patient was not under the influence of liquor, and five hours had elapsed since last taking food (breakfast). The purity of the nitrous oxide was tested shortly after the accident by the President of the Dental Association, Dr. Beers, who himself inhaled it from the same inhaler. The verdict of the jury was that the man died from syncope, caused by the administration of the gas, and they exonerated the dentist from blame.—*Druggists' Circular*, September, 1890.

Women Physicians in India.—From a parliamentary paper which has just been issued, we gather that a considerable number of native women have taken up the study of medicine in India. At the close of the session 1888-9 there were twenty-four female students at the Calcutta Medical College, fourteen at the Campbell Medical School, and five at the Cuttack Medical School. At Agra, during the year, seven young women received licenses to practice. At Lahore there were nineteen, and at Madras thirty-nine female medical students, one of the latter being the first to take the degree of M.D. at the Madras University. There were also female students at the Grant Medical College of Bombay, and at the Government Medical Schools at Poonah, Ahmedabad, and Hyderabad. The movement initiated, or at all events patronized by Lady Dufferin, is thus giving good fruit, and as the objections entertained by many over here to the practice of medicine by females do not apply in India, with its peculiar social conditions, this result must be a matter for congratulation.—*Medical Press*, September 3, 1890.

The County Dental Society of Philadelphia has adopted a protest against dentists being classified by the Census Bureau with manufacturers, and instructed its members to decline "to attempt the impossibility of making any returns" upon blanks under such classification.

